# Genomic Analysis of Carbon Monoxide Utilization and Butanol Production by *Clostridium carboxidivorans* Strain P7^T^

**DOI:** 10.1371/journal.pone.0013033

**Published:** 2010-09-27

**Authors:** Guillaume Bruant, Marie-Josée Lévesque, Chardeen Peter, Serge R. Guiot, Luke Masson

**Affiliations:** 1 Biotechnology Research Institute, National Research Council of Canada, Montreal, Quebec, Canada; 2 Département de Microbiologie et Immunologie, Université de Montréal, Montréal, Quebec, Canada; University of Hyderabad, India

## Abstract

Increasing demand for the production of renewable fuels has recently generated a particular interest in microbial production of butanol. Anaerobic bacteria, such as *Clostridium* spp., can naturally convert carbohydrates into a variety of primary products, including alcohols like butanol. The genetics of microorganisms like *Clostridium acetobutylicum* have been well studied and their solvent-producing metabolic pathways characterized. In contrast, less is known about the genetics of *Clostridium* spp. capable of converting syngas or its individual components into solvents. In this study, the type of strain of a new solventogenic *Clostridium* species, *C. carboxidivorans*, was genetically characterized by genome sequencing. *C. carboxidivorans* strain P7^T^ possessed a complete Wood-Ljungdahl pathway gene cluster, involving CO and CO_2_ fixation and conversion to acetyl-CoA. Moreover, with the exception of an acetone production pathway, all the genetic determinants of canonical ABE metabolic pathways for acetate, butyrate, ethanol and butanol production were present in the P7^T^ chromosome. The functionality of these pathways was also confirmed by growth of P7^T^ on CO and production of CO_2_ as well as volatile fatty acids (acetate and butyrate) and solvents (ethanol and butanol). P7^T^ was also found to harbour a 19 Kbp plasmid, which did not include essential or butanol production related genes. This study has generated in depth knowledge of the P7^T^ genome, which will be helpful in developing metabolic engineering strategies to improve *C. carboxidivorans's* natural capacity to produce potential biofuels from syngas.

## Introduction

Due to the increasing consumption and fluctuating price of oil, the limited supply of fossil fuels and petroleum, and growing concern about global warming and greenhouse gases, in the last few decades, there has been an increased interest in the production of renewable fuels such as ethanol and butanol. Compared to ethanol, butanol's low volatility, low proneness to hydration and high energy content make it a second generation biofuel of choice which could progressively replace gasoline [1]. Production of biofuels, such as biobutanol, by microorganisms is not particularly common, however some anaerobic bacteria, such as *Clostridium* spp., can accomplish this naturally [2], [3], [4], [5], [6]. Industrial production of butanol by solvent-producing *Clostridium* spp. strains was one of the first large scale fermentation processes developed [5]. To date, four distinct species of Clostridia have been identified for industrial butanol production, with *C. acetobutylicum* and *C. beijerinckii* being the two most extensively studied [5], [7], [8]. To date, these two solvent-producing Clostridia use carbohydrates as a carbon feedstock for alcohol production via the acetone-butanol-ethanol (ABE) fermentation [5], [7], [8]. Production of this feedstock, either by extraction (corn, soybean, sugar cane or sugar beets) or from lignocellulosic materials poses both economical problems, such as high pre-treatment costs to convert cellulose into a usable form, and ethical problems, such as creation of new arable land (i.e. Amazon forest conversion to soybean production) or diversion of current arable land to biofuel feedstock versus food production.

To circumvent such problems, an alternative approach would be to convert organic biomass via gasification to basic single carbon and hydrogen gases, and to use the produced synthesis gas (syngas) as feedstock for the synthesis of higher chain biofuels such as butanol. Carbon monoxide (CO) and molecular hydrogen (H_2_), the essential components of syngas, can be used as substrates for microbial metabolism [9], [10]. Several microorganisms, mostly known as acetogens, have already been shown to produce biofuels and other products of interest through syngas or CO fermentation. Among those, some acetogenic Clostridia species have been characterized, such as *Butyribacterium methylotrophicum* which produces both ethanol and butanol from CO [10], [11], and *C. autoethanogenum* and *C. ljungdahlii* which both produce ethanol from CO and syngas [12], [13], [14]. Recently, a new solvent-producing *Clostridium* species, named *C. carboxidivorans*, was isolated from an agricultural settling lagoon after enrichment with CO [15]. This organism can ferment CO into a mixture of acetate, butyrate, ethanol and butanol [10], [15], [16].

Over the past decade, many studies have been performed on the genetics of solvent-producing *Clostridium* strains and on metabolic engineering strategies for increasing butanol production from carbohydrate sources with these organisms [17], [18], [19]. As a consequence, this has led to a better understanding of their solvent-producing metabolic pathways. In contrast to solvent-producing *Clostridium* spp. using carbohydrates to produce butanol, less is known about the genetics of *Clostridium* spp. capable of converting syngas into solvents. Due to the modest butanol production increases observed in previous metabolic engineering studies and the advantages of syngas versus carbohydrate fermentation (i.e. conversion of poorly degradable biomass sources, such as straw and wood, into solvents), it is important to obtain deeper insight into the metabolic pathways of solventogenic microorganisms such as *C. carboxidivorans*. In this study, we characterized the new solvent-producing *Clostridium* species, *C. carboxidivorans* strain P7^T^, by sequencing its genome and determining the initial levels of solvent production by CO fermentation. The genetic elements involved in this microorganism's metabolic pathways for CO utilization, acetate and butyrate as well as ethanol and butanol production, were compared with those from other solvent-producing Clostridia.

## Results

### *C. carboxidivorans* strain P7^T^ growth and VFA/solvent production under a CO atmosphere

**Figure 1:**
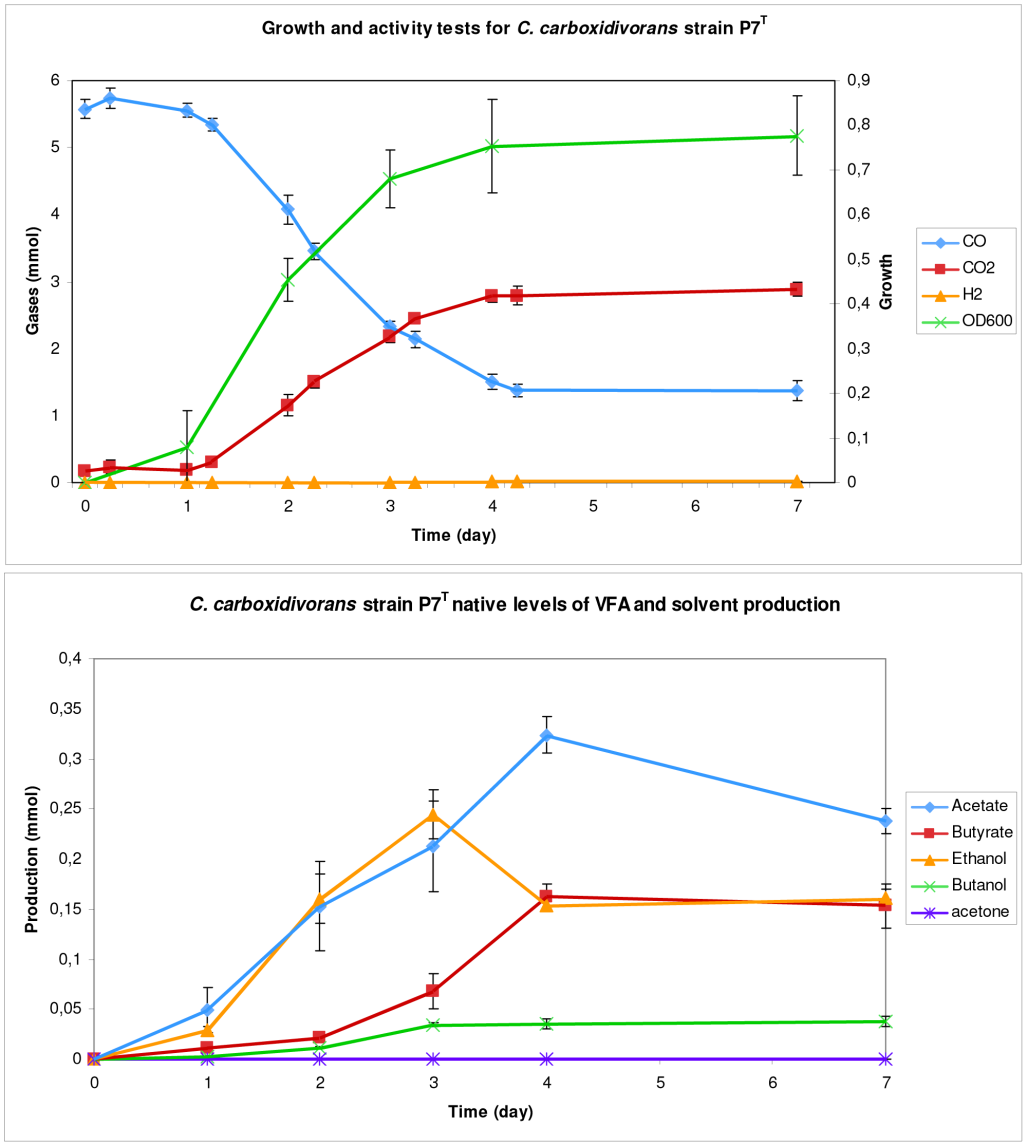
Growth and native levels of VFA and solvent production of *C. carboxidivorans* strain P7^T^. A) Strain P7^T^ growth (OD_600_), CO consumption and CO_2_ and H_2_ production. B) Strain P7^T^ native levels of VFA (acetate and butyrate) and solvents (ethanol, butanol and acetone) production.

Strain P7^T^ growth, CO consumption and gas production were monitored in parallel as shown in Figure 1A. After a 24 h lag phase, P7^T^ grew and reached a plateau (OD_600_ of 0.77) after four days of incubation at 37°C with an atmosphere of 100% CO. Bacterial growth was coupled with CO consumption and CO_2_ production. After four days of incubation, 73% of the CO initially present in the bottles was consumed. CO consumption and CO_2_ production stopped when strain P7^T^ reached stationary phase. At that point, only traces of H_2_ were detected.

P7^T^ volatile fatty acids (VFA) and solvent production was monitored by measuring the concentration of acetate, butyrate, ethanol, butanol and acetone every 24 h for four days and after seven days of incubation. As shown in Figure 1B, acetate, butyrate, ethanol and butanol appeared after 24 h of incubation whereas no acetone was evident after seven days of incubation. Acetate was the major product with 56.58 mmol produced per mol of CO consumed, followed by ethanol (38.21 mmol produced per mol of CO consumed), butyrate (36.45 mmol produced per mol of CO consumed) and butanol (8.91 mmol produced per mol of CO consumed).

### *C. carboxidivorans* strain P7^T^ genome general characteristics

Sequencing the genome of *C. carboxidivorans* strain P7^T^ generated 151 large contigs ranging from 500 to 215,023 bases with an estimated average of 40X sequence coverage. One additional 19,899 bp contig was identified as a potential plasmid sequence. P7^T^ consists of a 5.63 Megabase pair (Mbp) chromosome with a GC content of 29.7%, and a 19.9 Kbp plasmid with a slightly lower GC content of 27.5% (Table 1). Annotation and analysis of the sequencing data was initiated using the RAST prokaryotic genome annotation server [20]. The P7^T^ chromosome was predicted to possess 5325 coding sequences (CDSs) for protein and four rRNA sequences (23S, 16S and two 5S) as well as 49 tRNA genes. The potential 19 Kbp plasmid was determined to code for 22 CDSs (Table 1).

**Table 1:** Comparison of *C. carboxidivorans* strain P7^T^ general genomic features with those of the other sequenced solvent-producing *Clostridium*.

	C. car.	C. a.	C. b.	C. l.	C. cel.	C. t.	C. s.	C. p.
Genome size (bp)	5,650,800	4,132,880	6,000,632	4,630,065	4,068,724	3,843,301	4,662,871	4,847,594
GC content (%)	29.7	30.0	29.0	31.0	37.0	38.0	45.0	35.0
Plasmid (size)	1 (19,899 bp)	1 (192,000 bp)	0	0	0	0	0	0
CDSs	5347	3847	5020	4198	3390	3189	4154	3902
rRNA	4	33	43	27	24	12	18	25
tRNA	49	73	94	72	63	56	68	61

*C. car.*, Clostridium carboxidivorans strain P7^T^; *C. a.*, Clostridium acetobutylicum strain ATCC 824; *C. b.*, Clostridium beijerinckii strain NCIMB 8052; *C. l.*, Clostridium ljungdahlii strain DSM 13528; *C. cel.*, Clostridium cellulolyticum strain H10; *C. t.*, Clostridium thermocellum strain ATCC 27405; *C. s.*, Clostridium saccharolyticum strain WM1; *C. p.*, Clostridium phytofermentans strain ISDg.

**Figure 2:**
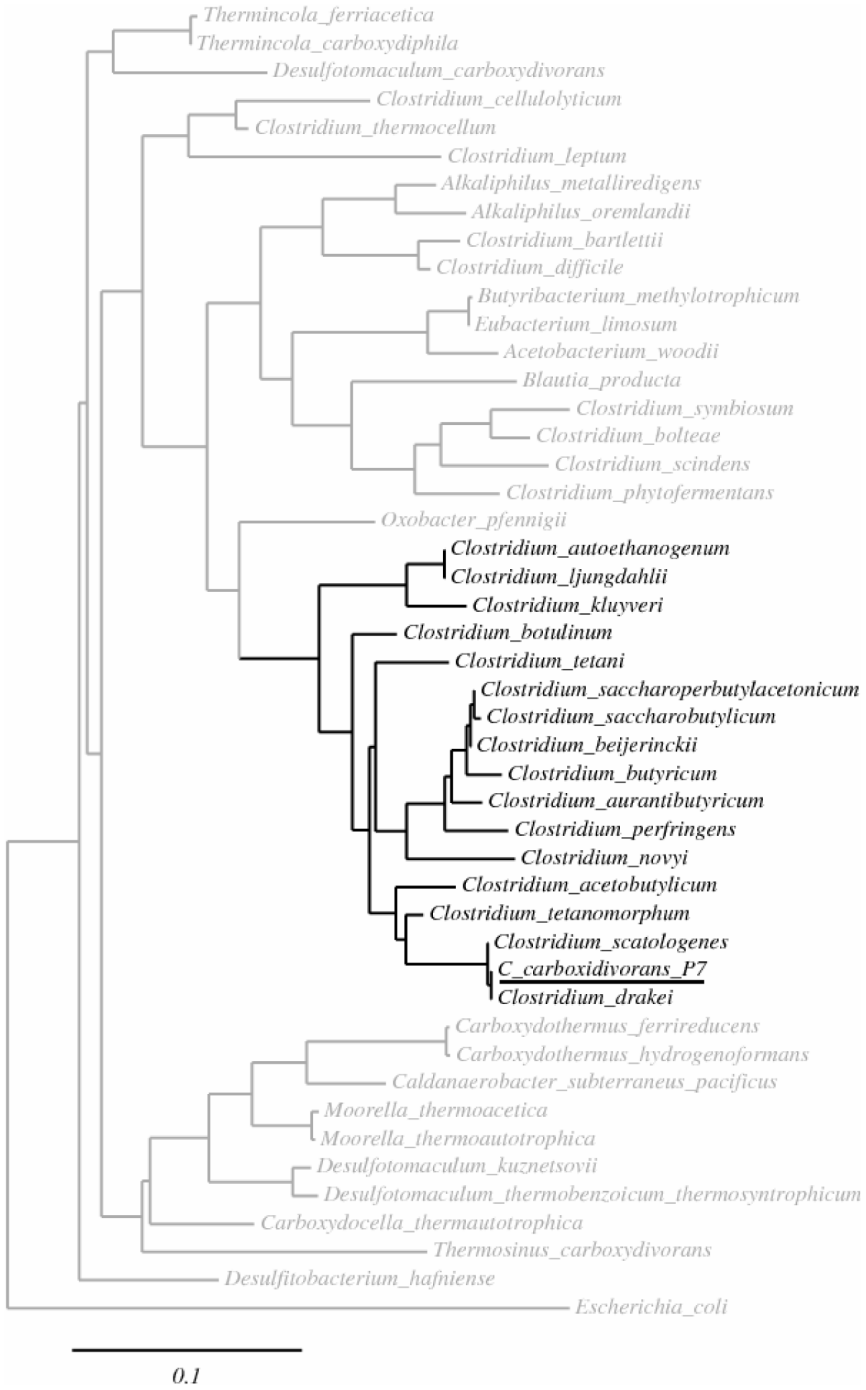
Phylogenetic tree. Phylogenetic tree based on complete 16S rRNA sequences from different acetogenic and/or solventogenic *Clostridium* species, and mesophilic and thermophilic acetogens. Horizontal bar represents sequence divergence. *C. carboxidivorans* strain P7^T^ is underlined.

Phylogenetic analyses were performed with the P7^T^ 16S rRNA sequence and complete 16S rRNA sequences from other members of the Clostridia class, and notably from the other known solventogenic *Clostridium* species. As shown in the generated tree (Figure 2), P7^T^ was most closely related to *C. drakei* and *C. scatologenes* species, as previously proposed by Liou *et al.* [15], but was also included in a phylogenetic group composed primarily of the solvent-producing *Clostridium* species such as *C. acetobutylicum*, *C. beijerinckii*, *C. saccharobutylicum*, *C. saccharoperbutylacetonicum*, *C. autoethanogenum* and *C. ljungdahlii*. Among the butanol-producing *Clostridium* species, P7^T^ was most closely related to *C. tetanomorphum* and *C. acetobutylicum*.

### Enzymes of the Wood-Ljungdahl pathway of CO(2) fixation

**Figure 3:**
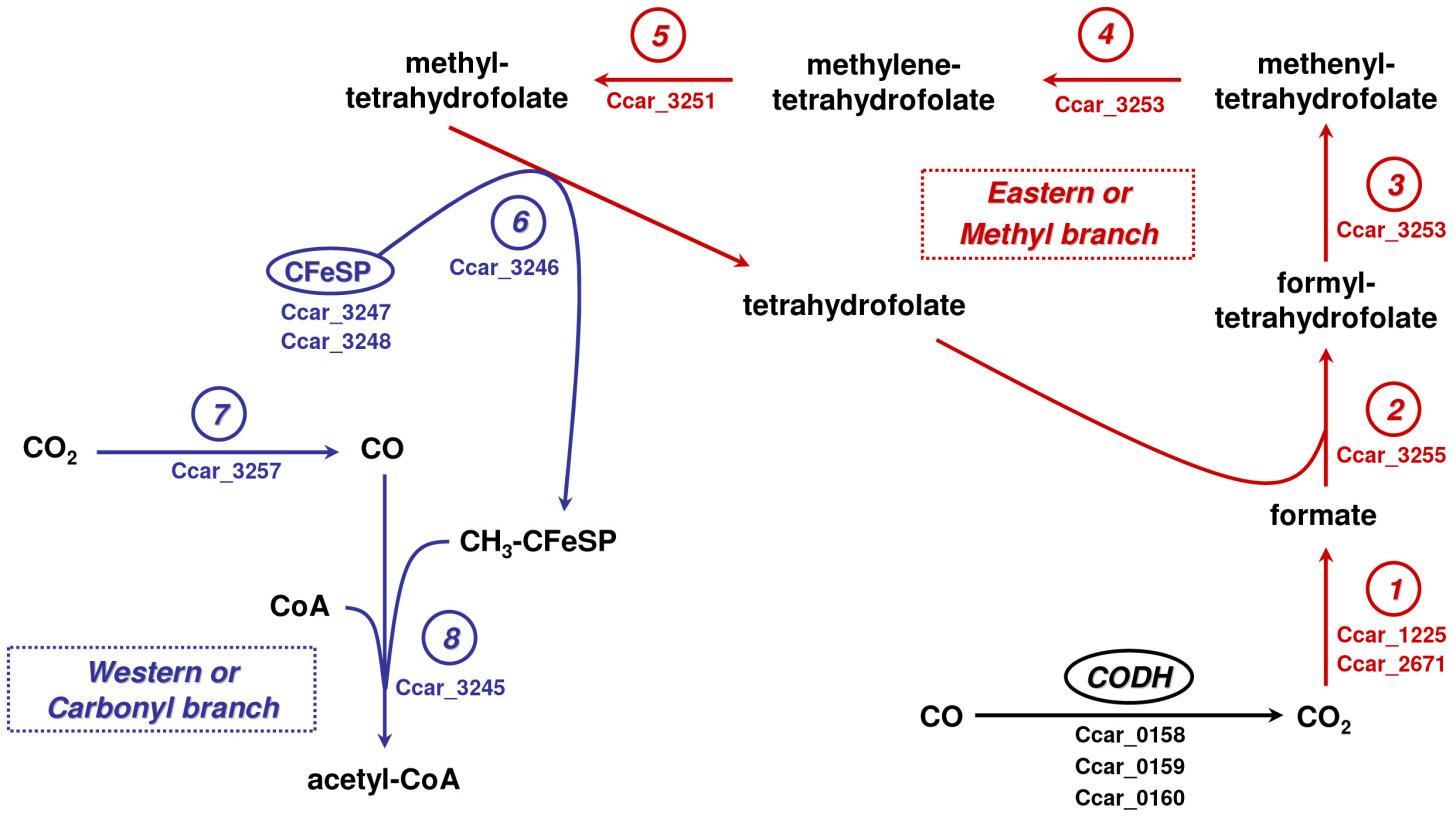
Wood-Ljungdahl pathway in *C. carboxidivorans* strain P7^T^. Wood-Ljungdahl pathway key enzymes and protein identified in *C. carboxidivorans* strain P7^T^. 1, formate dehydrogenase; 2, formate-tetrahydrofolate ligase; 3 and 4, bifunctional methenyl-tetrahydrofolate cyclohydrolase/methylene-tetrahydrofolate dehydrogenase (NADP+); 5, 5,10-methylene-tetrahydrofolate reductase; 6, 5-methyl-tetrahydrofolate:corrinoid iron-sulfur protein methyltransferase; 7, carbon monoxide dehydrogenase; 8, acetyl-CoA synthase; CFeSP, corrinoid iron-sulfur protein; CODH, additional carbon monoxide dehydrogenase complex. Reactions from the western branch are indicated in blue, those from the eastern branch are indicated in red. The corresponding genes in strain P7^T^ genome are indicated below the enzyme.

**Figure 4:**
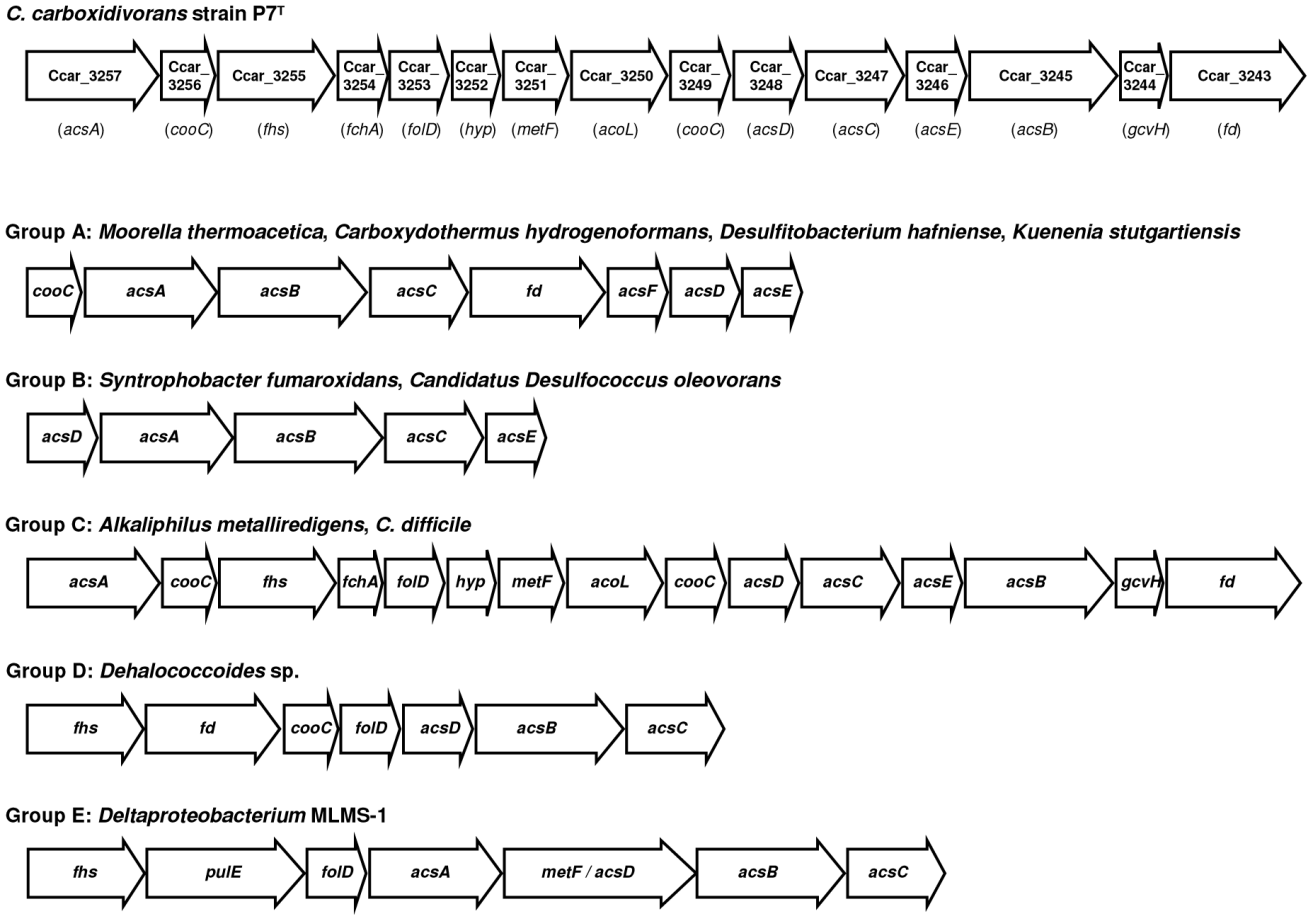
Wood-Ljungdahl pathway gene cluster in *C. carboxidivorans* strain P7^T^. Comparison between the Wood-Ljungdahl pathway gene cluster from *C. carboxidivorans* strain P7^T^ and those from the five groups described by Pierce *et al*. [23]. *cooC*, CODH accessory protein; *acsA*, CODH/ACS complex, CODH subunit; *acsB*, CODH/ACS complex, ACS subunit; *acsC*, corrinoid iron-sulfur protein large subunit; *fd*, ferredoxin; *acsF*, CODH accessory protein similar to CooC; *acsD*, corrinoid iron-sulfur protein small subunit; *acsE*, CODH/ACS complex, methyltransferase subunit; *fhs*, formyl-tetrahydrofolate synthase; *fchA*, formimido-tetrahydrofolate cyclodeaminase; *folD*, bifunctional methylene-tetrahydrofolate dehydrogenase/formyl-tetrahydrofolate cyclohydrolase; *hyp*, hypothetical protein; *metF*, methylene-tetrahydrofolate reductase; *acoL*, CODH/ACS complex, dihydrolipoamide dehydrogenase subunit; *gcvH*, glycine cleavage system H protein; *pulE*, ATPase.

Seventeen CDSs encoding all the enzymes and proteins of both eastern and western branches of the Wood-Ljungdahl pathway were characterized in the P7^T^ chromosome (Table S1). Key enzymes of this pathway and their corresponding CDS identified in strain P7^T^ are presented in Figure 3. The two first CDSs, Ccar_1225 and Ccar_2671, encoded respectively a selenocysteine-containing formate dehydrogenase H and a formate dehydrogenase accessory protein, both involved in the conversion of CO_2_ to formate [21]. Those two CDSs were found elsewhere on the chromosome away from the 15 others (CDSs Ccar_3243 to Ccar_3257) that clustered together, strictly identical in composition and organization to the Wood-Ljungdahl pathway gene clusters of *C. difficile* strain 630 [22] and *Alkaliphilus metalliredigens* strain QYMF (Figure 4). High levels of sequence identity were observed between the gene and protein sequences of these three clusters (Table S1). The eastern branch of the *C. carboxidivorans* strain P7^T^ Wood-Ljungdahl pathway was composed of all the enzymes involved in the conversion of formate to methyl-tetrahydrofolate [21], including a formate-tetrahydrofolate ligase involved in the conversion of formate to 10-formyl-tetrahydrofolate, a bifunctional methylene-tetrahydrofolate dehydrogenase/methenyl-tetrahydrofolate cyclohydrolase involved in the conversion of 10-formyl-tetrahydrofolate to 5,10-methylene-tetrahydrofolate, and a 5,10-methylene-tetrahydrofolate reductase which converts 5,10-methylene-tetrahydrofolate to 5-methyl-tetrahydrofolate. The western branch of strain P7^T^ Wood-Ljungdahl pathway was composed of all the enzymes involved in the conversion of CO_2_ to acetyl-CoA [21], including a corrinoid iron-sulfur protein (CFeSP), a 5-methyl-tetrahydrofolate:CFeSP methyltransferase which demethylates methyl-tetrahydrofolate to tetrahydrofolate, a CO dehydrogenase (CODH)/acetyl-CoA synthase (ACS) complex with a catalytic subunit CooS involved in the conversion of CO_2_ to CO and an ACS subunit responsible for the conversion of CO to acetyl-CoA, and two CODH accessory proteins. The five last CDSs composing this cluster were predicted to encode a formimino-tetrahydrofolate cyclodeaminase involved in the conversion of 5,10-methenyl-tetrahydrofolate to 10-formyl-tetrahydrofolate, a dihydrolipoamide dehydrogenase, a glycine cleavage system H protein, an electron transfer protein and a zinc-finger protein containing the methylene-tetrahydrofolate reductase C terminal domain.

In addition to the similarities observed with *C. difficile* strain 630 and *A. metalliredigens* strain QYMF, the 17 proteins from P7^T^'s Wood-Ljungdahl pathway were highly similar to proteins composing Wood-Ljungdahl pathways from *C. ljungdahlii* strain DSM 13528; ranging from 47% to 91% identity or 71% to 96% similarity, and *C. bartlettii* strain DSM 16795; ranging from 41% to 82% identity or 59% to 88% similarity. Relatively high similarities were also observed with proteins of Wood-Ljungdahl pathways from other Clostridia. Interestingly, proteins from the western branch shared relatively high identities (34% to 66%) with proteins of Wood-Ljungdahl pathways from clostridia species such as *Moorella thermoacetica*, *Carboxydothermus hydrogenoformans* and *Desulfitobacterium hafniense*, which belong to the Wood-Ljungdahl pathway gene cluster group A, as described by Pierce *et al.* [23] (Figure 4).

Finally, three CDSs, Ccar_0158 to Ccar_0160, encoding the enzymes of an additional CO dehydrogenase complex (Figure 3), have been characterized in the P7^T^ chromosome. Those CDSs were clustered together on the chromosome in the same organization as observed for the CO dehydrogenase complex recently characterized in *C. ljungdahlii* strain DSM 13528 and involved in the conversion of CO to CO_2_ [24]. High similarities, ranging from 77% to 86% identity (89% to 94% similarity), were observed between the proteins of those two clusters.

### Genes encoding enzymes of VFA and solvent metabolic pathways

**Figure 5:**
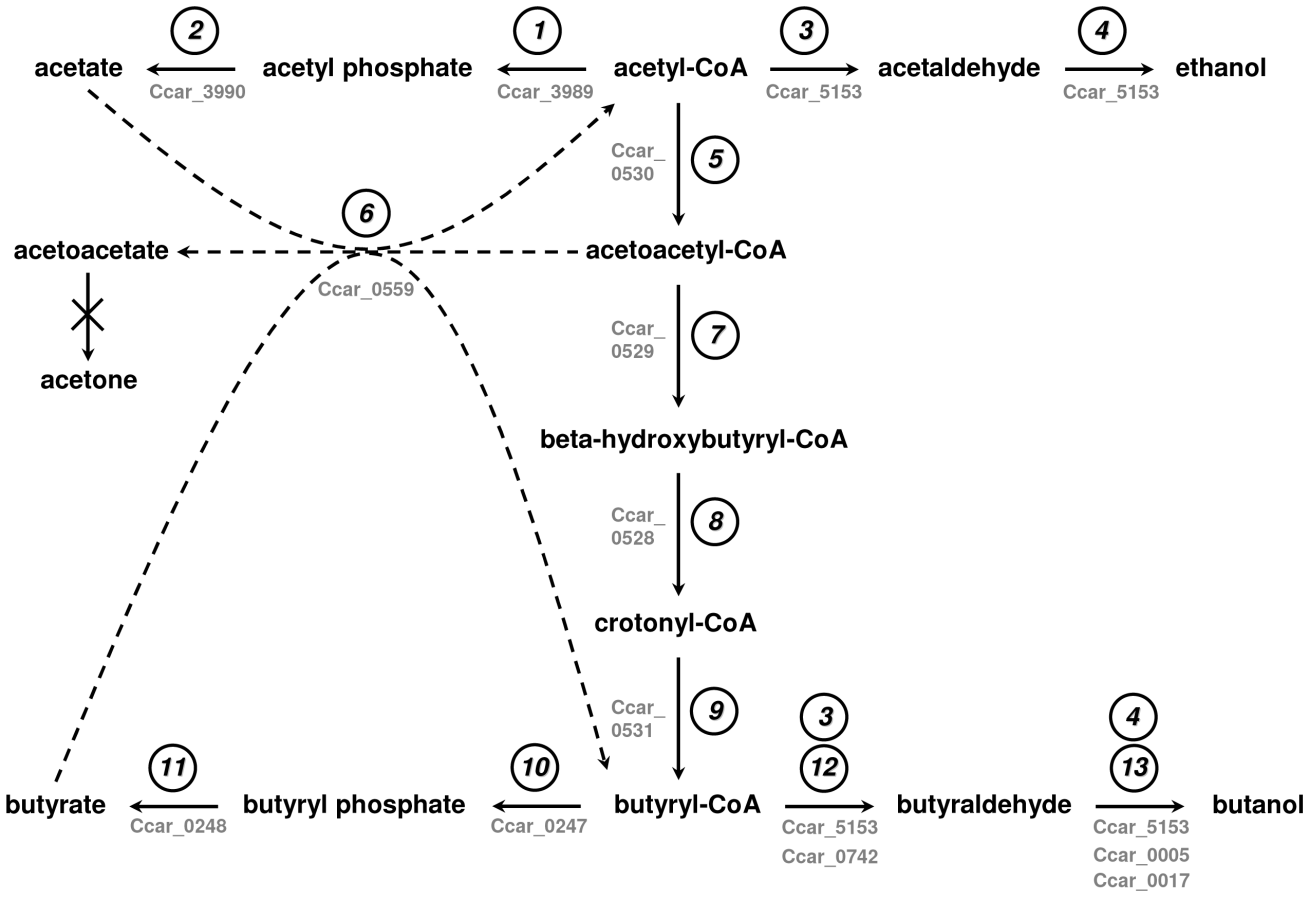
Metabolic fermentation pathways of acetyl-CoA in *C. carboxidivorans* strain P7^T^. 1, phosphate acetyltransferase; 2, acetate kinase; 3 and 4, bifunctional acetaldehyde/alcohol dehydrogenase; 5, acetyl-CoA acetyltransferase; 6, coenzyme A transferase; 7, 3-hydroxybutyryl-CoA dehydrogenase; 8, 3-hydroxybutyryl-CoA dehydratase; 9, butyryl-CoA dehydrogenase; 10, phosphate butyryltransferase; 11, butyrate kinase; 12, aldehyde dehydrogenase; 13, NADH-dependent butanol dehydrogenase. The corresponding genes in *C. carboxidivorans* strain P7^T^ genome are indicated below or next to each arrow. Dashed arrows indicate that the reactions may not occur in *C. carboxidivorans* strain P7^T^.

**Figure 6:**
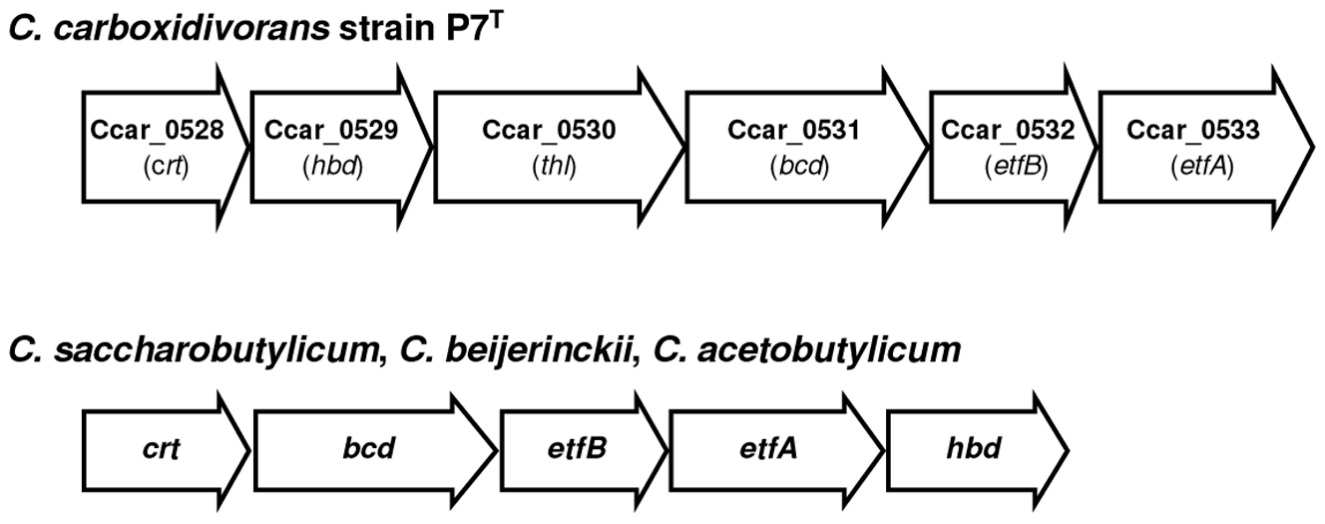
Comparison of the *bcs* gene clusters from *C. carboxidivorans* strain P7^T^ and other butanol-producing *Clostridium*. Organization and gene composition of the *bcs* cluster from strain P7^T^ and from *C. saccharobutylicum*, *C. beijerinckii* and *C. acetobutylicum* as described by Berezina *et al.* [38]. *crt*, crotonase; *hbd*, 3-hydroxybutyryl-CoA dehydrogenase; *thl*, acetyl-CoA acetyl transferase; *bcd*, butyryl-CoA dehydrogenase; *etfB*, electron transport protein, subunit B; *etfA*, electron transport protein, subunit A.

The P7^T^ sequence data was examined for enzymes involved in acetate and butyrate production, as well as those involved in ethanol and butanol production. As shown in Figure 5, VFA and solvent metabolic pathways in P7^T^ were similar to the ABE fermentation pathways of *C. acetobutylicum* strain ATCC 824 and *C. beijerinckii* strain NCIMB 8052. High levels of identity were observed between both gene and protein sequences from those strains (Table S2). Two adjacent CDSs, separated by a 16 bp spacer, were determined to belong to the acetate production pathway. CDS Ccar_3989 was predicted to encode a phosphate acetyltransferase involved in the conversion of acetyl-CoA to acetyl phosphate, and CDS Ccar_3990 encoded an acetate kinase responsible for the formation of acetate from acetyl phosphate. Eight CDSs were predicted to be involved in the butyrate production pathway. Six of them, Ccar_0528 to Ccar_0533, code for enzymes involved in the conversion of acetyl-CoA to butyryl-CoA and were clustered together on the chromosome (Figure 6). CDS Ccar_0528 was predicted to encode a 3-hydroxybutyryl-CoA dehydratase responsible for the conversion of beta-hydroxybutyryl-CoA to crotonyl-CoA. CDS Ccar_0529, separated from Ccar_0528 by a 73 bp spacer, was predicted to encode a 3-hydroxybutyryl-CoA dehydrogenase involved in the conversion of acetoacetyl-CoA to beta-hydroxybutyryl-CoA. CDS Ccar_0530, which was 69 bp downstream from Ccar_0529, was predicted to encode for an acetyl-CoA acetyltransferase which converts acetyl-CoA to acetoacetyl-CoA. CDS Ccar_0531, separated from Ccar_0530 by a 168 bp spacer, encodes for a butyryl-CoA dehydrogenase that converts crotonyl-CoA to butyryl-CoA. The last two CDSs of this cluster were 12 bp downstream from Ccar_0531 and were separated from each other by an 18 bp spacer. CDSs Ccar_0532 and Ccar_0533 were predicted to encode the beta and the alpha subunits of an electron transfer flavoprotein, respectively. It was of interest to note that a second copy of this cluster was found elsewhere in the chromosome. In addition to differences in nucleotide sequences (75% to 82% identity between corresponding CDSs), this second cluster, composed by CDSs Ccar_0550 to Ccar_0557, also included an additional truncated 3-hydroxybutyryl-CoA dehydratase encoding CDS (Ccar_0554). This feature (additional incomplete or modified cluster) has already been observed in other *Clostridium* species, such as *C. beijerinckii*, *C. botulinum* A and *C. difficile*. The last two CDSs predicted to be involved in the butyrate production pathway were adjacent on the chromosome, separated from each other by a 58 bp spacer. CDS Ccar_0247 encoded a phosphate butyryltransferase responsible for the conversion of butyryl-CoA to butyryl phosphate, and Ccar_0248 was predicted to encode a butyrate kinase involved in the conversion of butyryl phosphate to butyrate. As for VFA, solvents metabolic pathways in *C. carboxidivorans* strain P7^T^ were similar to the ABE fermentation pathways of *C. acetobutylicum* strain ATCC 824 and *C. beijerinckii* strain NCIMB 8052 (Figure 5) and high similarities were observed between both nucleotide and protein sequences from those strains (Table S2). One CDS, Ccar_5153, present in the chromosome in two adjacent copies, was characterized for the ethanol and butanol production pathways and encoded a bifunctional alcohol/acetaldehyde dehydrogenase involved in the conversion of acetyl-CoA to ethanol and butyryl-CoA to butanol. In addition to the CDSs encoding the enzymes involved in the conversion of acetyl-CoA to butyryl-CoA, three other CDSs were characterized to be involved in the butanol production pathway of *C. carboxidivorans* strain P7^T^. CDS Ccar_0742 was predicted to encode a CoA-acylating aldehyde dehydrogenase involved in the conversion of butyryl-CoA to butyraldehyde. The two other CDSs, Ccar_0005 and Ccar_0017, were both predicted to encode a NADH-dependent butanol dehydrogenase responsible for the conversion of butyraldehyde to butanol.

In addition to the similarities observed with *C. acetobutylicum* strain ATCC 824 and *C. beijerinckii* strain NCIMB 8052, all the proteins of *C. carboxidivorans* strain P7^T^ VFA and solvent pathways also possessed relatively high similarities, ranging from 51% to 81% identity, with proteins from other Clostridia, such as *C. bartlettii*, *C. botulinum* A, *C. butyricum*, *C. tyrobutyricum*, *C. saccharolyticum*, *C. difficile*, *C. hydrogenoformans* and *A. metalliredigens*. Proteins involved in *C. carboxidivorans* strain P7^T^ butanol production, and particularly proteins responsible for the conversion of butyryl-CoA to butanol, possessed high similarities with the two other butanol-producing *Clostridium* species *C. saccharobutylicum* and *C. saccharoperbutylacetonicum*. The CoA-acylating aldehyde dehydrogenase encoded by Ccar_0742 possessed 53% identity (71% similarity) with the CoA-acylating aldehyde dehydrogenase from *C. saccharobutylicum* and 53% identity (71% similarity) with the butyraldehyde dehydrogenase from *C. saccharoperbutylacetonicum*. The NADH-dependent butanol dehydrogenases encoded by Ccar_0005 and Ccar_0017 possessed respectively 55% and 72% identity (75% and 86% similarity) with the NADH-dependent butanol dehydrogenases from *C. saccharoperbutylacetonicum* and *C. saccharobutylicum*.

Finally, for the acetone production pathway, several copies of a CoA transferase encoding CDS, Ccar_0559, were characterized in the chromosome of *C. carboxidivorans* strain P7^T^. In addition to the high percentage of identity observed with the CoA transferases from *C. beijerinckii* strain NCIMB 8052 (Table S2), relatively high similarities, ranging from 51% to 73% identity (69% to 88% similarity), were also observed with CoA transferases from other Clostridia, including *C. botulinum*, *A. metalliredigens* and *C. butyricum*. In contrast, much lower similarities were observed with CoA transferases encoded by the *ctfA* and *ctfB* genes from *C. acetobutylicum*, with the CtfA transferase being 30% identical (44% similar) and the CtfB transferase being 26% identical (41% similar) to the CoA transferase encoded by CDS Ccar_0559 (Table S2). In addition, no acetoacetate decarboxylase encoding CDS, also involved in the acetone production pathway, was characterized in *C. carboxidivorans* strain P7^T^ genome. This absence was confirmed by PCR experiments performed with degenerated primers designed from a consensus sequence of acetoacetate decarboxylase encoding genes from several solvent-producing Clostridia (data not shown).

### Characterization of plasmid sequences

**Figure 7:**
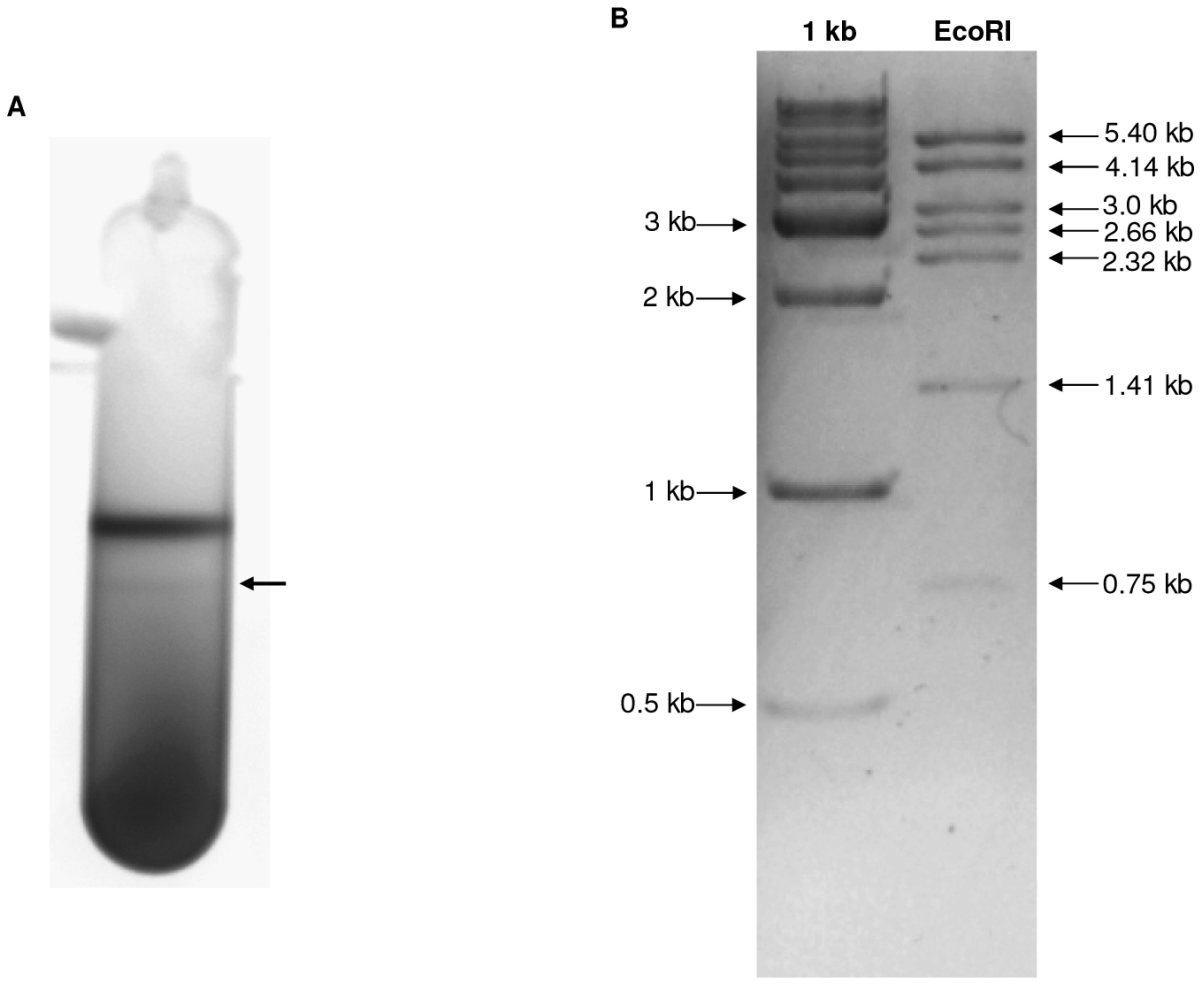
Confirmation of the presence of a plasmid in *C. carboxidivorans* strain P7^T^. A) Isolation of plasmid p19 from strain P7^T^ by standard cesium chloride gradient. Visualization of the two bands of DNA with UV illumination. Arrow indicate the lower band which contains the closed circular plasmid DNA. B) Digestion of the plasmid DNA by *Eco*RI. 1 kb ladder was used. Sizes are indicated on the left (ladder) and on the right (plasmid DNA).

Sequence data indicated the potential presence of a 19 Kbp plasmid in *C. carboxidivorans* strain P7^T^ due to the higher frequency of reads from a particular contig and its slightly lower GC content. To confirm the existence of this plasmid, clarified cytosolic lysates from P7^T^ were subjected to cesium chloride gradients. A high density lower band was determined to be present in the gradient (Figure 7A) and was subsequently extracted, purified and characterized. An *Eco*RI restriction digestion of the plasmid band should have produced eight bands however only seven bands of the predicted size were visualized (Figure 7B). The missing 233 bp band, presumably due to its small size, could not be visualized on the gel. Nonetheless, the digest confirmed the presence of an autonomous extra-chromosomal replication unit. This P7^T^ plasmid, which was named p19 in relation to its size, was relatively small as compared to the plasmids described in other Clostridia which have an average size of 83.5 kb (ranging from 7.9 kb to 270 kb), but possessed a similar GC content (27.5% versus 26.5% in general from other Clostridial plasmids). Sequence analysis of p19 revealed the presence of 22 CDSs, with a majority (15) potentially encoding proteins with either hypothetical or unknown functions. No significant similarity in function and in CDS organization was found when compared to plasmids from other Clostridia.

Among the seven plasmid CDSs encoding proteins with known functions, three, Ccar_4290, Ccar_4298 and Ccar_4300, were predicted to encode phage related proteins showing low to high similarities (23% to 67% identity, 38% to 80% similarity) with phage related proteins from other Clostridia, including *C. botulinum*, *C. butyricum*, *C. kluyveri*, *C. difficile*, *C. cellulovorans* and *C. perfringens*. CDS Ccar_4298 represents a putative phage integrase/recombinase family protein, which showed 51% identity (72% similarity) with an integrase from *C. papyrosolvens* and lower similarities (29% to 31% identity) with phage integrase family proteins from other Clostrida such as *C. botulinum* and *C. cellulovorans*. A second plasmid CDS was also predicted to encode an integrase/recombinase protein. CDS Ccar_4291 encodes for a protein showing 60% identity (78% similarity) with the integrase/recombinase XerD encoded by plasmid pCLJ2 from *C. botulinum* Ba4, and 37% to 45% identity (60% to 66% similarity) with integrase/recombinase proteins from other Clostridia, including *C. novyi*, *C. cellulovorans*, *C. perfringens*, and *C. difficile*. CDS Ccar_4289 was predicted to encode the replication protein of the plasmid. This protein showed 80% identity (89% similarity) with the replication protein (orf5) from *C. botulinum* strain Bf, and lower similarities (22% to 35% identity, 39% to 58% similarity) with the replication proteins from various plasmids described in other Clostridia, including plasmid pE88 from *C. tetani*, plasmids pCLG1 and pCBF from *C. botulinum*, plasmids pSM101B and pBCNF5603 from *C. perfringens* and plasmid pCD6 from *C. difficile*. The two last CDSs characterized in P7^T^ plasmid, Ccar_4283 and Ccar_4286, were predicted to encode proteins involved in chromosome partitioning. The chromosome segregation protein SMC encoded by Ccar_4283 showed 66% identity (81% similarity) with proteins encoded by plasmids pCKL1 and pCKL555A from *C. kluyveri*, and lower similarities (29% to 31% identity, 52% to 56% similarity) with proteins encoded by plasmids from *C. butyricum* and *C. perfringens*. Finally, CDS Ccar_4286 encoded a cobyrinic acid a,c-diamide synthase, which showed relatively high similarities (39% to 45% identity, 62% to 67% similarity) with the sporulation initiation inhibitor protein Soj from other Clostridia, including *C. hathewayi*, *C. asparagiforme*, *C. difficile*, *C. nexile* and *C. thermocellum*, as well as from several other microorganisms such as *Listeria* sp., *Bacillus* sp. and *Rickettsia* sp.

## Discussion

In this study, we characterized the type strain of a new solvent-producing *Clostridium* species, *C. carboxidivorans* strain P7^T^. It has been described as a mesophilic carboxydotrophic microorganism, which has a primarily acetogenic metabolism and is capable of converting CO into a mixture of VFA (acetate and butyrate) and solvents (ethanol and butanol) [15], [16], [25]. We have demonstrated the capacity of P7^T^ to produce acetate, butyrate, ethanol and butanol from CO, but not acetone unlike other solventogenic organisms. As described previously [15], butanol production by P7^T^ under CO was lower than acetate and ethanol, however, contrary to previous studies, we observed that the major end-product was acetate and not ethanol, and that butyrate was not detected at trace levels but at the same level as ethanol (Figure 1). The 100% CO atmosphere used in this study, as compared to CO/N_2_/CO_2_ (70∶24∶6) used by Liou *et al.* [15] for P7^T^ growth could possibly explain the discrepancies observed with previous data.

To identify the sets of genes responsible for CO utilization and for acetate, butyrate, ethanol and butanol production, the P7^T^ genome was sequenced and partially annotated. *C. carboxidivorans* possesses, with *C. beijerinckii*, one of the largest genomes among the sequenced solvent-producing *Clostridium*, and is the only sequenced Clostridial genome, with the notable exeption of *C. acetobutylicum*, to possess a plasmid (Table 1). Both nucleotide and protein sequence comparisons with other solvent-producing Clostridia were performed. We first confirmed the phylogenetic close relatedness of P7^T^ with the two acetogens *C. drakei* and *C. scatologenes*, as previously observed by Liou *et al.* [15], by analyses of Clostridia 16S rRNA sequences. In addition, we showed that P7^T^ was part of a phylogenetic group comprised mostly of known solvent-producing *Clostridium* species, including *C. acetobutylicum*, *C. beijerinckii*, *C. ljungdahlii*, *C. saccharobutylicum*, *C. saccharoperbutylacetonicum*, *C. autoethanogenum*, *C. aurantibutyricum*, and *C. tetanomorphum*, and was phylogenetically closest to *C. tetanomorphum* and *C. acetobutylicum* within this group (Figure 2). This result suggests that solventogenesis in *Clostridium* species could be a phylogenetic trait, whereas carboxydotrophy would not. Indeed, further genomic analysis of P7^T^ suggests that *Clostridium* carboxydotrophy is more a metabolic trait rather than a phylogenetic one. We delineated the genetic determinants of the P7^T^ metabolic pathways for CO utilization and characterized a complete Wood-Ljungdahl pathway gene cluster in its chromosome. This pathway is normally found in a large range of microbial classes, primarily archea methanogens and bacterial acetogens, and allows microorganisms to fix CO and CO_2_ and subsequently convert them to acetyl-CoA by the reductive acetyl-CoA pathway [21], [26]. The pathway is divided in two parts, named the eastern and the western branches. Genes encoding enzymes from the eastern branch are involved in the conversion of formate to methyl-tetrahydrofolate. Those genes are ubiquitous and generally scattered around the chromosome. In contrast, genes encoding enzymes from the western branch, called the core set of the Wood-Ljungdahl pathway gene cluster, are co-localized on the chromosome. All the enzymes composing the Wood-Ljungdahl pathway have now been well studied and well characterized, notably in the model acetogenic bacterium *Moorella thermoacetica* [21], [23]. According to Pierce *et al.* [23], Wood-Ljungdahl gene clusters can be classified into five groups depending on their gene content and organization. The P7^T^ Wood-Ljungdahl pathway gene cluster was strictly identical in gene content and organization (Figure 4), including the highest nucleotide and protein sequences identities, with Wood-Ljungdahl clusters from *C. difficile* strain 630 and *A. metalliredigens* strain QYMF, which belongs to Pierce *et al.* classification group C [23], and from two other Clostridia, the solvent-producing *C. ljungdahlii* strain DSM 13528 [24] and the acetogen *C. bartlettii* strain DSM 16795. These latter four Clostridia were genetically closely related but were not all placed in the same rDNA phylogenetic group as P7^T^ (Figure 2). In addition to this complete Wood-ljungdahl pathway gene cluster, an additional CO dehydrogenase complex, highly similar to the one characterized in the *C. ljungdahlii* strain DSM 13528 responsible for the conversion of CO to CO_2_ [24], was found in the P7^T^ chromosome. Finally, genes encoding a selenocysteine-containing formate dehydrogenase, highly similar to the one characterized in *C. ljungdahlii* strain DSM 13528 responsible for the conversion of CO_2_ to formate [24], and its accessory protein FdhD, required for formate dehydrogenase activity, were also found, indicating that P7^T^ possesses all the genetic determinants necessary for the conversion of CO to methyl-tetrahydrofolate via CO_2_ and formate (eastern branch of the Wood-Ljungdahl pathway) and of CO_2_ to acetyl-CoA via CO (western branch of the Wood-Ljungdahl pathway) [21], [26]. Acetyl-CoA could be subsequently converted to acetate, butyrate, ethanol and butanol through fermentation, presumably ABE fermentation.

Solventogenic Clostridia capable of converting a wide range of carbon sources into VFA and solvents through ABE fermentation, and particularly butanol-producing Clostridia such as *C. acetobutylicum*, *C. beijerinckii* and *C. pasteurianum*, exhibit very similar metabolic pathways [27], [28], [29]. Those pathways are now well documented and all the key enzymes involved in the ABE fermentation routes of butanol-producing Clostridia have been well characterized [30]. As we have confirmed the production of acetate, butyrate, ethanol and butanol from CO in addition to the absence of acetone production by P7^T^, all the genes encoding the ABE metabolic pathway enzymes responsible for the conversion of acetyl-CoA to acetate and acetyl-Coa to butyrate were examined (Figure 5). In both cases, high identities in both nucleotide and protein sequences were observed with the ABE metabolic pathways elements from other solvent-producing Clostridia. In addition, the phosphate acetyltransferase (*pta*) and acetate kinase (*ack*) genes, as well as the phosphate butyryltransferase (*ptb*) and butyrate kinase (*buk*) genes were localized in tandem in the P7^T^ chromosome, in the same manner as previously described for *ptb* and *buk* in *C. acetobutylicum* strain ATCC 824 [31] and *C. beijerinckii* strain NCIMB 8052 [32], as well as for genes *pta* and *ack* for *C. acetobutylicum* strain ATCC 824 [33]. In those strains, both sets of genes exist in tandem on the chromosome and form operons with *ptb* preceding *buk* and *pta* preceding *ack* [31], [32], [33].

Genetic determinants of ABE metabolic pathways for ethanol and butanol production were also characterized in P7^T^ (Figure 5). A gene encoding a bifunctional alcohol/acetaldehyde dehydrogenase highly similar to the AdhE proteins from *C. acetobutylicum* and *C. beijerinckii* was found in the chromosome. This bifunctional P7^T^ enzyme, as previously described for *C. acetobutylicum*, may be responsible for the conversion of acetyl-CoA to ethanol via acetaldehyde and of butyryl-CoA to butanol via butyraldehyde [34], [35], [36]. P7^T^ also possessed a chromosomal gene encoding a CoA-acylating aldehyde dehydrogenase highly similar to ones from *C. beijerinckii*, *C. saccharobutylicum* and *C. saccharoperbutylacetonicum*, which have been shown to be responsible for the conversion of butyryl-CoA to butyraldehyde and which distinguished those butanol-producing strains from *C. acetobutylicum* [37]. As P7^T^ also possessed genes encoding two butanol dehydrogenases highly similar to the ones responsible for the conversion of butyraldehyde to butanol in *C. acetobutylicum* and *C. beijerinckii*, it is likely that in *C. carboxidivorans*, as described for *C. acetobutylicum* by Durre *et al.*, 1995 the bifunctional alcohol/acetaldehyde dehydrogenase is responsible for the onset of solventogenesis and the CoA-acylating aldehyde dehydrogenase and the two butanol dehydrogenases ensure continued butanol production [35]. In addition to those genetic determinants, P7^T^ also possessed all the genes involved in butanol formation found in the canonical ABE metabolic pathway [30]. It was interesting to note that although P7^T^ genes involved in the conversion of acetyl-CoA to butyryl-CoA were grouped together on the chromosome (Figure 6), as described in *C. saccharobutylicum*, *C. acetobutylicum* and *C. beijerinckii* strains [38], those genes in P7^T^ were not found in the same organization as in the *bcs* operons and an additional gene, which encoded an acetyl-CoA acetyltransferase, was present (Figure 6). Nonetheless, in the P7^T^ cluster, the genes encoding the two subunits of the electron transfer flavoprotein were located immediately downstream of the gene encoding the butyryl-CoA dehydrogenase, as described in *C. saccharobutylicum*, *C. acetobutylicum* and *C. beijerinckii bcs* clusters [38]. This may imply that in *C. carboxidivorans*, the activity of the butyryl-CoA dehydrogenase requires the electron transfer flavoprotein and that the genes encoding those proteins should be expressed simultaneously, as has been previously shown in *C. acetobutylicum* [33], [39].

With the exception of the acetone production pathway, P7^T^ possessed similar ABE metabolic pathways for acetate, butyrate, ethanol and butanol production than those present in the other solvent-producing *Clostridium*. Contrary to the butanol-producing species *C. acetobutylicum*, *C. beijerinckii*, *C. saccharobutylicum* and *C. saccharoperbutylacetonicum*, in which the acetone production genetic determinants are a small cluster of three genes *adc*, *ctfA* and *ctfB* grouped together in the *adc* operon [38], neither acetoacetate decarboxylase encoding gene nor *ctfA* and *ctfB* genes were present in P7^T^. This is consistent with the absence of detection of acetone among the fermentation end-products of *C. carboxidivorans* strain P7^T^. Surprisingly, the uptake of acetate and, to a lower degree, butyrate, was observed after several days of growth (Figure 1), indicating a potentially active CoA transferase encoded by Ccar_0559 (Figure 5). However, in such a situation, the uptake of acetate and butyrate by CoA transferase would be concomitant with an accumulation of acetoacetate, since no acetone is formed. It is more probable that the P7^T^ CoA transferase may not be involved but rather the uptake of acetate and butyrate occurs via the reversal of the formation pathways as has been previously described by metabolic flux analysis with *C. acetobutylicum* [40]. Further work is required to verify this hypothesis.

In summary, the solventogenic bacterium *C. carboxidivorans* type strain P7^T^ was biochemically and genetically characterized in this study and was found to possess all the genetic determinants for CO utilization and for acetate, butyrate, ethanol and butanol production but lacked the acetone production pathway. In addition, P7^T^ also harbours a plasmid but unlike pSOL1, which harbours genes involved in solvent production in the butanol producing *C. acetobutylicum* strain ATCC 824 [41], this plasmid does not include either essential or butanol production related genes. Overall, in depth knowledge of the P7^T^ genome will be helpful in developing metabolic engineering strategies to improve *C. carboxidivorans's* natural capacity to produce potential biofuels from syngas.

## Materials and Methods

### Strain, medium and growth conditions

*C. carboxidivorans* type strain P7^T^ (DSM 15243) was obtained from the German Collection of Microorganisms and Cell Cultures (Deutsche Sammlung von Mikroorganismen und Zellkulturen GmbH (DSMZ), Braunschweig, Germany). Strain P7^T^ was grown under strictly anaerobic conditions in a minimal medium derived from a previously described acetogen medium [42] containing per liter: 1 g of NH_4_Cl; 0.8 g of NaCl; 0.1 g of KCl; 0.1 g of KH_2_PO_4_; 0.2 g of MgSO_4_, 7H_2_O; 0.04 g of CaCl_2_, H_2_O; 5 mL of a 4% cysteine-sulfide solution; 10 mL of a vitamin solution [25]; 10 mL of a trace metal solution [25]; and 0.5 g of yeast extract (Difco Laboratories, Detroit, MI, USA). Cultures were performed under an atmosphere of 100% CO (Praxair Canada Inc., Saint-Laurent, QC, Canada) and a pressure of 1 atmosphere (atm), at 37°C in 120 mL serum bottles containing 20 mL of minimal medium, with resazurin solution (0.1%) used as a redox indicator. The bottles were placed under a constant agitation of 100 rpm. The initial pH of the medium was adjusted to 6.0 with a 1 N NaOH solution and 2-(N-morpholino)ethanesulfonic acid (MES) (1.0 g/L) (Sigma Chemical Co., St. Louis, MO, USA) was used as a buffer. Growth was monitored by following the optical density at 600 nm (OD_600_) using a NanoDrop™ 1000 Spectrophotometer (Thermo Fisher Scientific, Wilmington, DE, USA).

### Analytical methods

Three serum bottles containing 20 mL of minimal medium were inoculated with *C. carboxidivorans* strain P7^T^ (10% v/v) which had reached log phase growth, and incubated at 37°C under agitation. Biochemical tests were performed during a seven day period. After 10 minutes at 37°C to allow for gas equilibrium, a sample (200 µL) of the headspace was taken for initial measurements of H_2_, CO_2_ and CO concentrations. The 200 µl were then injected into a HP 6890 gas chromatograph (Hewlett Packard, Palo Alto, CA, USA) equipped with a thermal conductivity detector and a 5 m×2.1 mm Carboxen-1000 column (Supelco, Bellafonte, CA, USA), with argon as carrier gas. The column temperature was held at 60°C for 7 min and increased to 225°C at a rate of 60°C per minute. H_2_, CO_2_ and CO concentrations were then determined in the same way after 6, 24, 30, 48, 54, 72, 78, 96 and 102 hrs of incubation. A final measurement was performed after seven days of incubation.

Culture supernatants were also analyzed every 24 h for four days and after seven days of incubation, for the presence of VFA and solvents (butanol, ethanol and acetone). Product concentrations were determined by gas chromatography with an Agilent 6890N gas chromatograph (Agilent Technologies, Wilmington, DE, USA) equipped with a flame ionization detector of 2 mL. For measurements of VFA, 0.2 µL of a sample fortified at a ratio of 1∶1 (v/v) using an internal standard of iso-butyric acid dissolved in 6% formic acid, were directly injected into a 1 m×2 mm, 60–80 mesh Carbopack C glass column (Supelco) coated with 0.3% Carbowax 20 M and 0.1% H_3_PO_4_. The column was held at 130°C for 4 min and helium, used as carrier gas, was injected at a rate of 20 mL/min. The injector and the detector were both maintained at 200°C. For solvent measurements, 100 µL of liquid sample were transferred in a 20 mL headspace vial crimped with a Teflon coated septum. Before injection, the vial was heated at 80°C for 2 minutes. One mL of the headspace was automatically injected into a 30 m×530 µm×2 µm DB-ACL2 capillary column (Agilent J&W GC column, Agilent Technologies). The column was held at 40°C for 10 minutes. Helium was used as carrier gas at a head pressure of 5 psi. The injector, under a split ratio of 5∶1, and the detector were maintained at 200°C and 250°C, respectively.

### DNA isolation

Total DNA was isolated from *C. carboxidivorans* strain P7^T^ using the MO BIO UltraClean™ Microbial DNA Isolation Kit (MO BIO Laboratories Inc., Carlsbad, CA, USA) according to manufacturer's instructions. In order to increase yields, a heating step of 10 min at 70°C was performed before the mechanical lysis step of the manufacturer's protocol. Quantification of DNA and purity assessment were performed using the *A*260/*A*280 ratio determined by a NanoDrop™ 1000 Spectrophotometer. The quality of the extracted DNA was also verified by electrophoresis in a 0.8% agarose gel followed by visualization with UV illumination after ethidium bromide staining.

Plasmid DNA was isolated by standard cesium chloride gradient centrifugation using ethidium bromide for DNA detection. Gradients were centrifuged at 45,000 rpm for at least 36 h at room temperature. The two bands of DNA, the upper one containing both genomic and uncoiled plasmid DNA and the lower one containing closed circular plasmid DNA, were then visualized with UV illumination, extracted and purified.

### Genome sequencing, annotation and analysis

The genome of *C. carboxidivorans* strain P7^T^ was sequenced at the Génome Québec Innovation Centre (McGill University and Génome Québec Innovation Centre, Montréal, QC, Canada) using a Roche 454 Life Sciences Genome Sequencer FLX (Roche 454 Life Sciences technology, Branford, CT, USA). The shotgun library was prepared with 5 µg of *C. carboxidivorans* total DNA. All general aspects of library construction, sequencing and sequence assemblies were performed at the Génome Québec Innovation Centre. Annotation and subsequent analysis of the *C. carboxidivorans* strain P7^T^ genome was performed using the Rapid Annotation Subsystem Technology (RAST) server version 2.0 [20] and the Basic Local Alignment Search Tool (BLAST) at the National Center for Biotechnology Information. Phylogenetic analyses were performed using the web-based phylogenetic tools at http://www.Phylogeny.fr [43] and 16S rRNA sequences from several Clostridia obtained from the Ribosomal Database site (http://rdp.cme.msu.edu/index.jsp) [44]. Phylogenetic trees were generated using the Maximum Likelihood method (100 bootstrap value) included in the PhyML 3.0 software of the Phylogeny.fr platform.

### Nucleotide sequences accession numbers

The nucleotide sequences described in this study have been deposited in the GenBank database under accession numbers HM590559 to HM590571 and HQ201313.

## Supporting Information

Table S1 Comparison of key enzyme sequences within the Wood-Ljungdahl pathways from *C. carboxidivorans* strain P7^T^, *C. difficile* strain 630 and *A. metalliredigens* strain QYMF.
(0.07 MB DOC)

Table S2 Sequence comparisons of key enzymes of VFA and solvent production pathways in *C. carboxidivorans* strain P7^T^ and related enzymes from two butanol-producing species, *C. acetobutylicum* and *C. beijerinckii*.
(0.07 MB DOC)
