# Supplementary material for: Genomic Analysis of Carbon Monoxide Utilization and Butanol Production by Clostridium carboxidivorans Strain P7T
Source: PLoS One. 2010 Sep 27;5(9):e13033. doi: 10.1371/journal.pone.0013033 (PMC2946384; doi:10.1371/journal.pone.0013033)
Supplement: Table S1 — Comparison of key enzyme sequences within the Wood-Ljungdahl pathways from C. carboxidivorans strain P7T, C. difficile strain 630 and A. metalliredigens strain QYMF. (0.07 MB DOC) [file pone.0013033.s001.doc]

| **Table S1. Comparison of key enzyme sequences within the Wood-Ljungdahl pathways from *C. carboxidivorans* strain P7^T^, *C. difficile* strain 630 and *A. metalliredigens* strain QYMF.** | | | | |
| --- | --- | --- | --- | --- |
| ***C. carboxidivorans* strain P7^T^ enzyme (encoding gene)** | ***C. difficile* strain 630 enzyme (encoding gene)** | **Protein sequence identity/similarity (%)** | ***A. metalliredigens* strain QYMF enzyme (encoding gene)** | **Protein sequence identity/similarity (%)** |
| formate dehydrogenase H, selenocysteine-containing (Ccar_1225) | formate dehydrogenase H (CD3317 / *fdhF*) | 77 / 86 | molybdopterin oxidoreductase (Amet_4098) | 34 / 47 |
|  |  |  |  |  |
| formate dehydrogenase chain D (Ccar_2671) | formate dehydrogenase accessory protein FdhD (CD3316 / *fdhD*) | 36 / 56 | formate dehydrogenase accessory protein (Amet_1000) | 35 / 53 |
|  |  |  |  |  |
| electron transfer protein (Ccar_3243) | iron-sulfur protein (CD0730) | 62 / 81 | ferredoxin (Amet_2030) | 68 / 85 |
|  |  |  |  |  |
| glycine cleavage system H protein (Ccar_3244) | glycine cleavage system protein H (CD0729 / *gcvH*) | 66 / 81 | glycine cleavage system H protein (Amet_2029) | 68 / 83 |
|  |  |  |  |  |
| CO dehydrogenase/acetyl-CoA synthase, acetyl-CoA synthase subunit (Ccar_3245) | bifunctional acetyl-CoA decarbonylase/synthase complex, subunit alpha/beta (CD0728) | 73 / 86 | CO dehydrogenase/acetyl-CoA synthase complex, beta subunit (Amet_2028) | 72 / 84 |
|  |  |  |  |  |
| 5-methyl-tetrahydrofolate:corrinoid iron-sulfur protein methyltransferase (Ccar_3246) | CO dehydrogenase/acetyl-CoA synthase complex, methyltransferase (CD0727) | 72 / 87 | dihydropteroate synthase (Amet_2027) | 71 / 86 |
|  |  |  |  |  |
| acetyl-CoA synthase corrinoid iron-sulfur protein, large subunit (Ccar_3247) | acetyl-CoA decarbonylase/synthase complex, gamma subunit (CD0726) | 64 / 80 | CO dehydrogenase/acetyl-CoA synthase complex, delta subunit (Amet_2026) | 67 / 81 |
|  |  |  |  |  |
| acetyl-CoA synthase corrinoid iron-sulfur protein, small subunit (Ccar_3248) | acetyl-CoA decarbonylase/synthase complex, delta subunit (CD0725) | 68 / 78 | CO dehydrogenase/acetyl-CoA synthase complex, delta subunit (Amet_2025) | 64 / 79 |
|  |  |  |  |  |
| CO dehydrogenase accessory protein CooC (Ccar_3249) | CO dehydrogenase/acetyl-CoA synthase complex, nickel-inserting subunit (CD0724) | 63 / 83 | cobyrinic acid a,c-diamide synthase (Amet_2024) | 70 / 81 |
|  |  |  |  |  |
| dihydrolipoamide dehydrogenase (Ccar_3250) | CO dehydrogenase/acetyl-CoA synthase complex, dihydrolipoyl dehydrogenase subunit (CD0723) | 66 / 79 | dihydrolipoamide dehydrogenase (Amet_2023) | 63 / 81 |
|  |  |  |  |  |
| 5,10-methylene-tetrahydrofolate reductase (Ccar_3251) | methylene-tetrahydrofolate reductase (CD0722) | 71 / 83 | methylene-tetrahydrofolate reductase (Amet_2022) | 76 / 87 |
|  |  |  |  |  |
| zinc-finger protein (Ccar_3252) | hypothetical protein (CD0721) | 69 / 83 | conserved hypothetical protein (Amet_2021) | 64 / 83 |
|  |  |  |  |  |
| bifunctional methylene-tetrahydrofolate dehydrogenase/methenyl-tetrahydrofolate cyclohydrolase (Ccar_3253) | FolD bifunctional protein (CD0720 / *folD*) | 70 / 84 | methylene-tetrahydrofolate dehydrogenase (Amet_2020) | 67 / 82 |
|  |  |  |  |  |
| formimino-tetrahydrofolate cyclodeaminase (Ccar_3254) | methenyl-tetrahydrofolate cyclohydrolase (CD0719 / *fchA*) | 75 / 87 | formiminotransferase-cyclodeaminase (Amet_2019) | 67 / 79 |
|  |  |  |  |  |
| formate-tetrahydrofolate ligase (Ccar_3255) | formate-tetrahydrofolate ligase (CD0718 / *fhs*) | 81 / 90 | formate-tetrahydrofolate ligase (Amet_2018) | 76 / 85 |
|  |  |  |  |  |
| CO dehydrogenase accessory protein CooC (Ccar_3256) | putative CO dehydrogenase accessory protein (CD0717 / *cooC*) | 70 / 89 | cobyrinic acid a,c-diamide synthase (Amet_2017) | 70 / 86 |
|  |  |  |  |  |
| CO dehydrogenase CooS subunit (Ccar_3257) | bifunctional CO dehydrogenase/acetyl-CoA synthase (CD0716 / *cooS*) | 60 / 75 | CO dehydrogenase, catalytic subunit (Amet_2016) | 61 / 78 |
