# Supplementary material for: Genomic Analysis of Carbon Monoxide Utilization and Butanol Production by Clostridium carboxidivorans Strain P7T
Source: PLoS One. 2010 Sep 27;5(9):e13033. doi: 10.1371/journal.pone.0013033 (PMC2946384; doi:10.1371/journal.pone.0013033)
Supplement: Table S2 — Sequence comparisons of key enzymes of VFA and solvent production pathways in C. carboxidivorans strain P7T and related enzymes from two butanol-producing species, C. acetobutylicum and C. beijerinckii. (0.07 MB DOC) [file pone.0013033.s002.doc]

| **Table S2. Sequence comparisons of key enzymes of VFA and solvent production pathways in *C. carboxidivorans* strain P7^T^ and related enzymes from two butanol-producing species, *C. acetobutylicum* and *C. beijerinckii*.** | | | | |
| --- | --- | --- | --- | --- |
| ***C. carboxidivorans* strain P7^T^ enzyme (encoding gene)** | ***C. acetobutylicum* strain ATCC 824 enzyme (encoding gene)** | **Protein sequence identity/similarity (%)** | ***C. beijerinckii* strain NCIMB 8052 enzyme (encoding gene)** | **Protein sequence identity/similarity (%)** |
| phosphate acetyltransferase (Ccar_3989) | phosphotransacetylase (CA_C1742 / *pta*) | 69 / 83 | phosphate acetyltransferase (Cbei_1164) | 71 / 83 |
|  |  |  |  |  |
| acetate kinase (Ccar_3990) | acetate kinase (CA_C1743 / *askA*) | 65 / 81 | acetate kinase (Cbei_1165) | 65 / 81 |
|  |  |  |  |  |
| phosphate butyryltransferase (Ccar_0247) | phosphate butyryltransferase (CA_C3076 / *ptb*) | 73 / 87 | phosphate butyryltransferase (Cbei_0203) | 67 / 84 |
|  |  |  |  |  |
| butyrate kinase (Ccar_0248) | butyrate kinase (CA_C3075 / *buk*) | 72 / 84 | butyrate kinase (Cbei_0204) | 68 / 80 |
|  |  |  |  |  |
| 3-hydroxybutyryl-CoA dehydratase (Ccar_0528) | crotonase (CA_C2712 / *crt*) | 61 / 77 | enoyl-CoA hydratase/isomerase (Cbei_0321) | 63 / 79 |
|  |  |  |  |  |
| 3-hydroxybutyryl-CoA dehydrogenase (Ccar_0529) | beta-hydroxybutyryl-CoA dehydrogenase (CA_C2708 / *hbd*) | 70 / 83 | 3-hydroxybutyryl-CoA dehydrogenase (Cbei_0325) | 73 / 85 |
|  |  |  |  |  |
| acetyl-CoA acetyltransferase (Ccar_0530) | acetyl-CoA acetyltransferase (CA_C2873) | 72 / 87 | acetyl-CoA acetyltransferase (Cbei_0411) | 76 / 87 |
|  | acetyl-CoA acetyltransferase (CA_P0078 / *thil*) | 72 / 83 |  |  |
|  |  |  |  |  |
| butyryl-CoA dehydrogenase (Ccar_0531) | butyryl-CoA dehydrogenase (CA_C2711 / *bcd*) | 79 / 89 | acyl-CoA dehydrogenase (Cbei_0322) | 72 / 86 |
|  |  |  |  |  |
| electron transfer flavoprotein, beta subunit (Ccar_0532) | electron transfer flavoprotein beta-subunit (CA_C2710 / *etfB*) | 79 / 88 | electron transfer flavoprotein, beta-subunit (Cbei_0323) | 69 / 80 |
|  |  |  |  |  |
| electron transfer flavoprotein, alpha subunit (Ccar_0533) | electron transfer flavoprotein alpha-subunit (CA_C2709 / *etfA*) | 69 / 84 | electron transfer flavoprotein, alpha-subunit (Cbei_0324) | 66 / 80 |
|  |  |  |  |  |
| alcohol / acetaldehyde dehydrogenase (Ccar_5153) | acetaldehyde-CoA/alcohol dehydrogenase (CA_P0035 / *adhe*) | 69 / 85 | acetaldehyde-CoA / alcohol dehydrogenase (Cbei_0305) | 74 / 87 |
|  | aldehyde-alcohol dehydrogenase (CA_P0162 / *adhe1*) | 69 / 83 |  |  |
|  |  |  |  |  |
| CoA-acylating aldehyde dehydrogenase (Ccar_0742) | acetaldehyde-CoA/alcohol dehydrogenase (CA_P0035 / *adhe*) | 33 / 51 | aldehyde dehydrogenase (Cbei_3832 / *ald*) | 53 / 70 |
|  | aldehyde-alcohol dehydrogenase (CA_P0162 / *adhe1*) | 30 / 50 |  |  |
|  |  |  |  |  |
| NADH-dependent butanol dehydrogenase (Ccar_0005) | acetaldehyde-CoA/alcohol dehydrogenase (CA_P0035 / *adhe*) | 41 / 58 | iron-containing alcohol dehydrogenase (Cbei_1722) | 77 / 88 |
|  | aldehyde-alcohol dehydrogenase (CA_P0162 / *adhe1*) | 36 / 57 |  |  |
|  | NADH-dependent butanol dehydrogenase A (CA_C3399 / *bdhA*) | 27 / 48 |  |  |
|  | NADH-dependent butanol dehydrogenase B (CA_C3298 / *bdhB*) | 26 / 45 |  |  |
|  |  |  |  |  |
| NADH-dependent butanol dehydrogenase A (Ccar_0017) | NADH-dependent butanol dehydrogenase A (CA_C3399 / *bdhA*) | 69 / 84 | iron-containing alcohol dehydrogenase (Cbei_2421) | 66 / 81 |
|  | NADH-dependent butanol dehydrogenase B (CA_C3298 / *bdhB*) | 65 / 85 |  |  |
|  |  |  |  |  |
| CoA transferase (Ccar_0559) | CoA transferase (CA_P0163 / *ctfA*) | 30 / 44 | CoA transferase (Cbei_3819) | 79 / 91 |
|  | CoA transferase (CA_P0164 / *ctfB*) | 26 / 41 |  |  |
